# Measuring the Compressibility of Cellulose Nanofiber-Stabilized Microdroplets Using Acoustophoresis

**DOI:** 10.3390/mi12121465

**Published:** 2021-11-27

**Authors:** Ksenia Loskutova, Karl Olofsson, Björn Hammarström, Martin Wiklund, Anna J. Svagan, Dmitry Grishenkov

**Affiliations:** 1 Department of Biomedical Engineering and Health Systems, Royal Institute of Technology, KTH-Flemingsberg, SE-141 57 Huddinge, Sweden; dmitryg@kth.se; 2 Department of Applied Physics, Royal Institute of Technology, KTH-Albanova, SE-106 91 Stockholm, Sweden; karlolo@kth.se (K.O.); bham@kth.se (B.H.); martin.wiklund@biox.kth.se (M.W.); 3 Department of Fibre and Polymer Technology, Royal Institute of Technology, KTH-Valhallavägen, SE-114 28 Stockholm, Sweden; svagan@kth.se

**Keywords:** acoustofluidics, ultrasound contrast agent, acoustic contrast factor, radiation force, compressibility, droplet vaporization, ultrasound-mediated drug delivery

## Abstract

Droplets with a liquid perfluoropentane core and a cellulose nanofiber shell have the potential to be used as drug carriers in ultrasound-mediated drug delivery. However, it is necessary to understand their mechanical properties to develop ultrasound imaging sequences that enable in vivo imaging of the vaporization process to ensure optimized drug delivery. In this work, the compressibility of droplets stabilized with cellulose nanofibers was estimated using acoustophoresis at three different acoustic pressures. Polyamide particles of known size and material properties were used for calibration. The droplet compressibility was then used to estimate the cellulose nanofiber bulk modulus and compare it to experimentally determined values. The results showed that the acoustic contrast factor for these droplets was negative, as the droplets relocated to pressure antinodes during ultrasonic actuation. The droplet compressibility was 6.6–6.8 $\times \;10^{-10}$ Pa${}^{-1}$, which is higher than for water ($4.4\times 10^{-10}$ Pa${}^{-1}$) but lower than for pure perfluoropentane ($2.7\times 10^{-9}$ Pa${}^{-1}$). The compressibility was constant across different droplet diameters, which was consistent with the idea that the shell thickness depends on the droplet size, rather than being constant.

## 1. Introduction

Drug delivery is a key aspect to consider when developing effective treatments for many types of diseases. Currently, there are many challenges to overcome to improve drug delivery in clinical settings. Firstly, some drugs are known to cause several severe side effects in humans, which is the case for many types of cancer drugs [1,2,3]. In other cases, for example, for neurological disorders such as Alzheimer’s and amyotrophic lateral sclerosis, the blood–brain barrier poses a challenge in delivering drugs to the central nervous system where the drug can effectively act [4,5]. Since many drugs are hydrophobic, effective drug delivery is still a major challenge to overcome for a more efficient treatment [6]. Encapsulating such drugs in a drug carrier, which releases the drug after a controlled external stimuli such as an ultrasound could both improve treatment efficacy and decrease side effects in patients [7].

Recently, a perfluoropentane (PFP) droplet with a cellulose nanofiber (CNF) shell stabilized via a Pickering mechanism was developed by Ghorbani et al. [8]. The perfluoropentane core inside the droplets is hydrophobic [9], which makes it possible to easily incorporate lipophilic drugs. CNFs have many hydroxyl groups present at the surface, which makes it easy to functionalize the droplet shell to target specific cell types [8,10]. Vaporized droplets were previously successfully imaged at clinically relevant acoustic pressure levels [11], which shows the potential to use these droplets not only as drug carriers but also for theranostics, which include imaging of drug delivery to the region of interest [12,13]. However, it is necessary to determine the mechanical properties of these droplets to develop an efficient ultrasound imaging sequence for visualization of droplets before and after drug delivery and to ensure that the drug is properly delivered to the region of interest.

Previous studies have successfully determined the acoustic impedance of suspended particles using acoustophoresis [14,15,16]. However, it has not been possible to determine mechanical properties separately as the acoustic contrast factor is dependent on both differences in density and compressibility between the particles and the suspension liquid. In this work, the compressibility of CNF-shelled perfluoropentane droplets was successfully determined using an acoustophoresis setup. Droplets were produced with a simple mixing step, and their compressibility and acoustic contrast factor were evaluated at three different acoustic pressures using Gorkov’s theory. Polyamide beads of known size were used for indirect pressure calibration. The droplet density was determined by estimating the CNF shell thickness with transmission electron microscopy. The bulk modulus for CNFs was estimated based on the experimentally determined droplet compressibility and compared to previously reported values. Finally, an assessment was made of the droplet compressibility values and their relation to shell thickness.

## 2. Theory

### 2.1. Acoustic Radiation Force

If particles suspended in a liquid are exposed to an ultrasonic standing wave (USW), they will be affected by an acoustic radiation force (ARF) which is a time-averaged effect caused by the scattering of induced acoustic waves by particles suspended in a liquid. The first studies of ARF were conducted by King in 1934, where he studied the forces acting on an incompressible particle in an inviscid fluid [17]. Yosioka and Kawasima included the effects of compressible particles [18]. Gorkov [19] combined the two previous works and generalized them to cover compressible particles suspended in an inviscid fluid with radius *a* much smaller than the wavelength of the acoustic wave λ (a<<λ). For the one-dimensional case, the acoustic radiation potential would then be equal to [20]

(1) $$U_{\mathrm{rad}}=\pi a^{3}\kappa_{o}p_{a}^{2}\times \left[\frac{f_{1}}{3}\cos^{2}\left(kx\right)-\frac{f_{2}}{2}\sin^{2}\left(kx\right)\right],$$

where $\kappa_{o}=1/\rho_{o}c_{o}^{2}$ is the compressibility of the suspension liquid, *k* is the wave number and $p_{a}$ is the pressure amplitude of the acoustic wave. The two acoustic coefficients, $f_{1}$ and $f_{2}$, come from scattering of the acoustic field and the translational movement of the particle, respectively. $f_{1}$ can be calculated using the compressibilities of the suspension liquid, $\kappa_{o}$, and the particle, $\kappa_{p}$:

(2a) $$f_{1}=1-\frac{\kappa_{p}}{\kappa_{o}}.$$

On the other hand, $f_{2}$ is based on the difference in densities, $\rho_{o}$ for the suspension liquid, and $\rho_{p}$ for the particle:

(2b) $$f_{2}=\frac{2(\rho_{p}-\rho_{o})}{2\rho_{p}+\rho_{o}}.$$

By combining these two coefficients, it is possible to determine the acoustic contrast factor Φ,

(3) $$\Phi =\frac{f_{1}}{3}+\frac{f_{2}}{2}=\frac{1}{3}\cdot \left(\frac{5\rho_{p}-2\rho_{o}}{2\rho_{p}+\rho_{o}}-\frac{\kappa_{p}}{\kappa_{o}}\right).$$

The acoustic radiation force $F_{\mathrm{rad}}$ can be calculated as the gradient of the acoustic radiation potential:

(4) $$\begin{array}{cc} F_{\mathrm{rad}} & =-\nabla U_{\mathrm{rad}} \end{array}$$

(5) $$\begin{array}{cc} & =4\pi a^{3}kE_{\mathrm{ac}}\Phi \sin\left(2kx\right), \end{array}$$

where $E_{\mathrm{ac}}=p_{a}^{2}/4\rho_{o}c_{o}^{2}$ is the acoustic energy density. The sign of Φ is what determines the direction of $F_{\mathrm{rad}}$, which in turn depends on whether the compressibility or density difference is larger and by how much. In the case of cells in acoustophoresis settings, Φ is often positive [14,21], which results in cells relocating to pressure nodes. However, the CNF-shelled droplets were previously reported to relocate to pressure antinodes even though the estimated droplet density is higher than for water [8], suggesting that the compressibility of CNF-shelled droplets is higher than the surrounding medium. If the acoustic energy density and the properties of the suspension liquid are known and the density of particles is established, it is possible to determine the particle compressibility.

### 2.2. Indirect Pressure Estimation

Determining the pressure inside a microchannel can be a challenge, since the microchannel is confined from all directions except for the outlets. Instead, an indirect measurement is taken where the pressure is estimated using the movements from suspended particles with known mechanical properties exposed to $F_{\mathrm{rad}}$. The particle velocity depends on $F_{\mathrm{rad}}$, the dynamic viscosity of the suspension liquid, η, the width of the microchannel *w*, and the position of the particle, *x* [20],

(6) $$v\left(x\right)=\frac{2\Phi ka^{2}E_{\mathrm{ac}}}{3\eta }sin\left(\frac{4\pi x}{w}\right).$$

The acoustic pressure $p_{a}$ can then be calculated by using $E_{\mathrm{ac}}$,

(7) $$p_{a}=2\sqrt{\rho_{o}c_{o}^{2}E_{\mathrm{ac}}}.$$

### 2.3. Theoretical Estimation of Compressibility

If the compressibility of the individual components of the droplets is known, it is possible to estimate the theoretical droplet compressibility. For an inhomogenous medium, the simplest model for describing the effective compressibility is the average of its components [22],

(8) $$\frac{1}{\kappa_{d}}=\left(1-\phi \right)\cdot \frac{1}{\kappa_{\mathrm{PFP}}}+\phi \cdot \frac{1}{\kappa_{\mathrm{CNF}}},$$

where ϕ is the volume fraction of the CNF shell, and $\kappa_{d}$, $\kappa_{\mathrm{PFP}}$, and $\kappa_{\mathrm{CNF}}$ are the compressibilities of the droplets, liquid PFP, and CNF, respectively. The volume of CNF, $V_{\mathrm{CNF}}$, can in turn be determined if the radius of the liquid PFP core *R* and the shell thickness *t* is known,

(9) $$V_{\mathrm{CNF}}=\frac{4\pi {\left(R+t\right)}^{3}}{3}-\frac{4\pi R^{3}}{3}.$$

The volume fraction of CNF ϕ is then determined by dividing $V_{\mathrm{CNF}}$ with the total droplet volume:

(10) $$\phi =1-\frac{R^{3}}{{\left(R+t\right)}^{3}}$$

## 3. Materials and Methods

### 3.1. Materials

PFP (99%) was ordered from Apollo Scientific (Stockport, UK). A 0.303 wt% CNF suspension with moderately cationic CNF was prepared according to a protocol described elsewhere [8,10]. In short, the suspension was produced by mixing a 1.3 wt% prepared CNF suspension with MilliQ water and sonicating the solution.

### 3.2. Droplet Preparation

**Figure 1:**
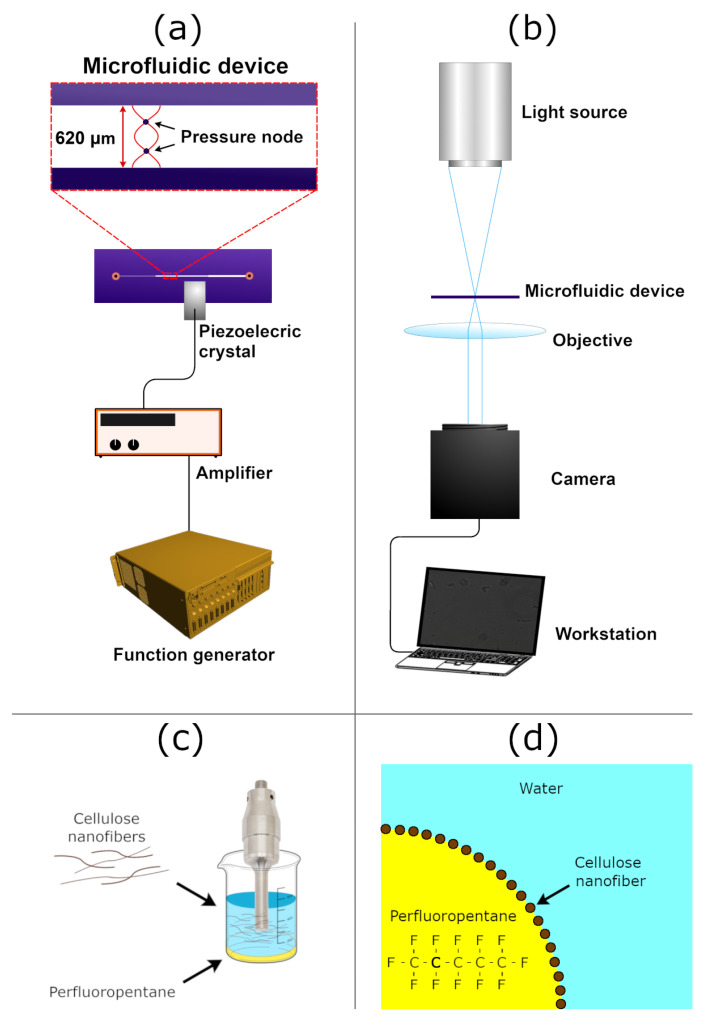
(**a**) The experimental setup depicting the microfluidic device used in the measurements. The piezoelectric crystal is glued to the top of the microfluidic device. The width of the microchannel is 620 µm. (**b**) The placement of the microfluidic device in the optical microscope during acoustophoresis and acoustic streaming measurements. (**c**) Simplified scheme depicting how the droplets are produced by sonication. (**d**) A simplified scheme of the droplets, where the CNFs are adsorbed at the interface between PFP and water.

The droplets were prepared using an easy mix and sonicate protocol described elsewhere [8] and visualized in Figure 1c. In short, 36.1174 grams of 0.303 wt% CNF suspension were mixed with 1.232 grams of PFP using a sonicator (Sonics Vibracell W750, Sonics and Materials Inc., Newton, CT, US). The suspension was stored in the refrigerator prior to use.

### 3.3. Droplet Characterization

#### 3.3.1. Optical Microscopy

Optical light microscopy (Axiovert 40 CFL, Zeiss, Germany) was used to determine the size distribution of droplets. A monocolor camera (BFS-U3-51S5M-C, FLIR, OR, USA) was used to capture the images. After tracking the particles, the diameter of each tracked droplet was manually measured in ImageJ.

#### 3.3.2. Transmission Electron Microscopy

The stock sample was diluted with water (1:99), placed on a carbon-coated copper grid and left to air dry for two hours. The grid with the sample was then placed in a JEM-2100F Field Emission Electron Microscope and imaged at 200 kV to acquire Transmission Electron Microscope (TEM) images. Images were collected using a TemCam-XF416 Complementary metal–oxide–semiconductor (CMOS) camera (Tvips, Germany).

#### 3.3.3. Field Emission Scanning Electron Microscopy

A Hitachi SEM S-4800 (Japan) was used at an accelerating voltage of 1 kV to acquire Scanning Electron Microscopy (SEM) images. A drop on the stock suspension of CNF-stabilized droplets was dropped on top of silicon wafers, allowed to dry at ambient conditions in the room, and then sputter-coated (Cressington 208HR sputter coater) with a Pt/Pd (60/40) coating (1.5 nm) prior to imaging.

### 3.4. Pressure Calibration

Polyamide beads with a diameter of 4.5 µm (EU-DFS-BMF-ver.1 for Flow Doppler Phantoms, Danish Phantom Design, Denmark) were used for pressure calibration of the microchannel device. Their physical properties are given in Table 1. The beads were injected into the microchannel shown in Figure 1a, and the ultrasound was activated when the flow was stopped. Images were recorded at 20 frames per second (FPS) for 30 s, generating a total of 600 images in each measurement. Only data points after ultrasound actuation were taken into account to determine the acoustic pressure.

A magnification of the signal of 40, 42, and 43 dB corresponded to 240, 325, and 360 kPa, respectively. The acoustic pressure of the USW was lower than the vaporization threshold for these droplets [8]. This means that the pressure amplitude was always below the vaporization pressure threshold for these type of droplets to avoid the creation of gas bubbles [8].

### 3.5. Acoustophoresis Tests

The device used for acoustophoresis measurements had a width of 620 µm and had a sandwich-like structure made out of glass–silicon–glass, visualized in Figure 1. The device was placed in an optical microscope (Axiovert 40 CFL, Zeiss, Germany) and connected to a monocolor camera (BFS-U3-51S5M-C, FLIR, OR, USA), a piezoelectric transducer, a function generator (DS345, Stanford Research Systems, CA, USA), and an RF amplifier (75A250, Amplifier Research, PA, USA). Only half of the microchannel was imaged due to limitations in the field of view of the optical microscope. The transmitted frequency was set to 2.40 MHz to match the wavelength criterion for a USW. First, the pressure calibration was performed following the procedure described in Section 3.4. The droplets were then introduced into the device in the same manner as the polyamide beads and exposed to ultrasound at acoustic pressures ranging from 240 to 360 kPa. The ultrasound was actuated one minute after introduction of droplets into the microcavity to allow them to sediment to the bottom. The images during acoustophoresis measurements at the two highest acoustic pressures were obtained at a frame rate of 20 FPS for 30 s. At the lowest acoustic pressure of 240 kPa, the images were obtained at a frame rate of 20 FPS for 30 s, and in the subsequent xml files, only one in five data points was taken into account due to low velocities. A suspension of droplets with a concentration of $24.9\times 10^{6}$ droplets/mL was used for the acoustophoresis tests.

### 3.6. Acoustic Streaming

The same device and ultrasound actuators were used as in the acoustophoresis tests. Two different types of beads were used to measure the effect of acoustic streaming: 1 µm (FluoSpheres™ Carboxylate-Modified Microspheres, 1.0 µm, crimson fluorescent (625/645)) and 0.5 µm (FluoSpheres Carboxylate-Modified Microspheres, 0.5 µm, yellow-green fluorescent (505/515)) from Invitrogen (Thermo Fisher Scientific, CA, USA). The concentrations of 1 µm and 0.5 µm beads used in these measurements were $1.00\times 10^{8}$ and $2.91\times 10^{9}$ beads/mL, respectively. The beads were introduced into the microchannel, and ultrasound actuation occurred when there was no flow present. The focus of the optical microscope was at the bottom of the microcavity, i.e., in the same focal plane as the location of the droplets in the acoustophoresis measurements. The movement of the beads was imaged using the same camera as in the acoustophoresis tests at 20 FPS for 30 seconds.

### 3.7. Image Analysis

The images from the acoustophoresis tests were first processed using TrackMate, a plugin developed for Fiji that converts images to an xml file with information about the position of each particle at each time point [26]. The images were converted from RGB to 32-bit gray value and analyzed using a Laplacian of Gaussian (LoG) detector and a Linear Assignment Problem (LAP) tracker. The xml files were then manually examined and the noise removed. Only data points after ultrasound actuation were taken into account for further analysis. A MatLab^®^ script was then used to fit the data to the known function depicted in Equation (6), and the compressibility and contrast factor were calculated. In the MatLab^®^ script used to calculate the droplet compressibility, it was assumed that the droplets had a constant density of 1618.6 kg/m^3^, and regions close to the cavity walls and center were excluded. Fiji was used to measure the shell thickness in transmission electron microscopy images (see Supporting Information). The images from acoustic streaming measurements were analyzed using OpenPIV, an open source package for Matlab^®^ [27]. The flow was studied by ensemble correlation.

## 4. Results and Discussion

### 4.1. Droplet Properties

**Figure 2:**
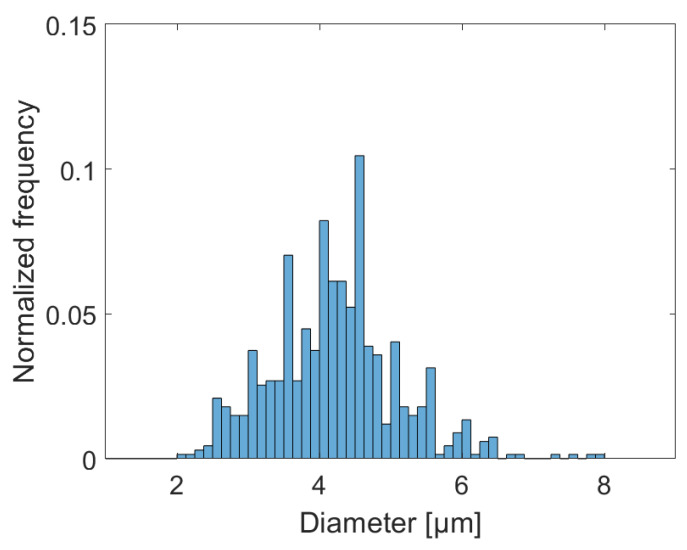
The number distribution of droplets obtained using optical microscopy at 20× magnification. The mean diameter for these droplets was measured at 4.2±0.9 µm.

The size distribution of droplets imaged during acoustophoresis measurements is visualized in Figure 2. The mean diameter for observed CNF-droplets was 4.2±0.9 µm.

### 4.2. Acoustic Contrast Factor of CNF-Shelled Droplets

All of the acoustic tests were performed below the vaporization threshold of the droplets, which was previously found to be 620 kPa [8]. The pressure of 620 kPa is within the range of common acoustic pressures used in acoustophoresis [28,29,30,31], and ensures that the droplets do not undergo acoustic droplet vaporization during measurements. Therefore, it is possible to state that the measured values of ϕ and compressibility are valid for liquid-filled non-vaporized CNF-shelled droplets.

**Figure 3:**
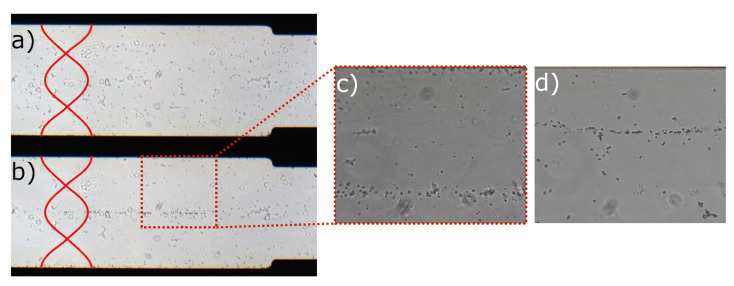
The spreading of droplets in the microchannel (**a**) before ultrasound actuation and (**b**) after ultrasound actuation. The red lines show the ultrasonic standing waves and the location of pressure nodes (where the lines cross each other) and pressure antinodes (where the red lines are at maximum distance from each other). (**c**) The droplets relocate to pressure antinodes, as compared to (**d**) polyamide beads in pressure nodes at the same conditions.

Figure 3 is an image taken during acoustophoresis measurements that shows the location of CNF-shelled droplets and polyamide particles before and after ultrasound actuation. The acoustic contrast factor for polyamide is positive [32], and, therefore, they relocate to pressure nodes as presented in Figure 3d. In opposition, droplets relocate to pressure antinodes, seen in Figure 3c. This suggests that CNF-shelled droplets have a negative acoustic contrast factor.

Table 2 presents the experimentally determined and the theoretically calculated acoustic contrast factor for droplets. The theoretical value of ϕ presented in Table 2 was determined based on droplet compressibility values and Equation (3). It was assumed that the shell thickness was t=6.7 nm and that the droplet radius was R=2.1 µm (or droplet diameter of 4.2 µm), where the value for the droplet radius comes from the mean value measured in the acoustophoresis setup. This gives a CNF volume fraction of 0.9511%, and consecutively a droplet density $\rho_{d}=1628.8$ kg/m^3^. Interestingly, we can see in Table 2 that the theoretical ϕ is in accordance with the experimental values, therefore confirming that this follows the predictions of Gorkov’s theory.

**Figure 4:**
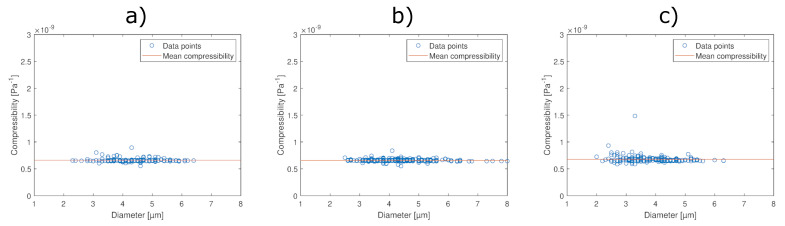
The compressibility of individual droplets at (**a**) 240 kPa, (**b**) 325 kPa, and (**c**) 360 kPa, where the density was set to constant based on the proportion of PFP and CNF in the preparation step and their respective densities presented in Table 1.

We can see in Table 2 that the acoustic contrast factor was larger at the highest acoustic pressure of 360 kPa compared to the values at 240 and 325 kPa. In addition, there was a higher variance in compressibility values at lower droplet diameters, as seen in Figure 4c. One probable explanation is that there are variations in the acoustic pressure in the direction of the microcavity, whilst it was assumed that the acoustic pressure was constant. This error would be more pronounced as the acoustic pressure in the microcavity increased.

Interestingly, although the contrast factor was negative, as shown by an earlier study [8], its absolute value was significantly lower than for previously studied microbubbles [30]. Oil droplets with negative ϕ reported by Wang et al. had a density that was lower than for water but a compressibility that was higher, yielding a relocation of oil droplets to pressure antinodes [28]. ϕ depends on both the difference in compressibility and density between the suspended droplets and surrounding medium. As can be seen from Table 1 and Table 2, the density and compressibility of droplets was higher than for water. This means that ϕ can be either negative or positive, depending on which of the differences are larger. Pure PFP, if stable in water, would have had an acoustic contrast factor of Φ=−4.7, while pure CNFs would have Φ≈ 0.9–1.2 based on the values presented in Table 1. The acoustic contrast factor for CNF-shelled lies within that range, suggesting that our estimations of the contrast factor are reasonable.

### 4.3. Compressibility of CNF-Shelled Droplets

The compressibility of Pickering-stabilized PFP-droplets was measured at three different acoustic pressures. Figure 4 shows the determined compressibility for each individual droplet at three different acoustic pressures. In addition, the experimentally determined droplet compressibility and the estimated values of CNF compressibility and bulk modulus are presented in Table 3. The compressibility of pure PFP ($27.7\times 10^{-10}$ Pa^−1^) [24], perfluorohexane ($16.93\times 10^{-10}$ Pa^−1^), and perfluoroheptane ($14.93\times 10^{-10}$ Pa^−1^) [33] has been found to be higher than for these droplets when comparing these values to what is presented in Table 3. The reason for the lower compressibility compared to perfluorocarbons is that the interface between PFP and the water is stabilized by CNF via a Pickering mechanism [10].

Figure 4 shows that the droplet compressibility is independent of diameter and constant. As we can see from Equation (8), the compressibility of droplets is dependent on the volume fraction of CNF in the droplet. If the shell thickness was constant, ϕ would decrease as the radius of the droplet increased (see Equation (10)). In this case, the droplet compressibility would increase with increasing droplet size. In opposition to this, we do not see any increase in compressibility, which is consistent with a constant ϕ.

**Figure 5:**
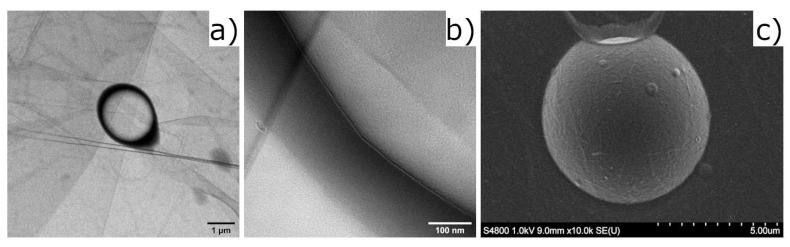
TEM image taken at 200 kV in cryo conditions of (**a**) zoomed out droplet, and (**b**) zoomed in on the border. The droplet exploded due to radiation damage from the electron beam, and, therefore, the shell is bent outwards from the border. (**c**) SEM image taken of the droplet, showing the coherent fibrous layer at the interface. The image is the original image produced by the software connected to the microscope, and the scale bar beneath the image is in micrometers.

CNF seemed to form a coherent monolayer at the PFP–water interface, shown in Figure 5c; however, the CNFs do have a distribution in thickness [10], and even larger individual fibrils could be seen in the suspension (see Figure S2 in Supporting Information). Only small CNFs are able to stabilize the interface in smaller droplets; however, both smaller and larger fibrils have this potential as the droplet size increases. Therefore, it could be possible that the larger fibrils form the shell of larger droplets, and that the volume fraction could be seen as constant.

The mechanical properties of CNF are highly dependent on parameters such as cellulose source, degree of polymerization, porosity, moisture content, nanofibril alignment, surface functionality, and the counterions present [34]. These parameters influence the intermolecular and intramolecular interactions, which is what in the end determine the tensile strength of the material [34]. The values for bulk modulus presented in Table 1 are probably an overestimation of the bulk modulus for our CNFs due to the drying process, as wetting the fibrils lowers the bulk modulus [23,34]. Because of this, it is more feasible to estimate the compressibility of CNF in the droplet shell and compare it to the values presented in Table 1.

To estimate the average of volume fraction of CNF in droplets, it was assumed that all CNFs present in the stock suspension were adsorbed at the interface between PFP and water. This gave us an average volume fraction of 8.8%, based on the data from Section 3.2. In acoustophoresis measurements, the density of the droplets was calculated by using values in Table 1 and assuming that the volume fraction of CNF was constant and equal to 8.8%,

(11) $$\phi \cdot \rho_{\mathrm{CNF}}+\left(1-\phi \right)\cdot \rho_{\mathrm{PFP}}=1618.56\;\;\mathrm{kg}/m^{3}.$$

By inserting the values for compressibility from Table 1 and from the acoustophoresis measurements into Equation (8), it is possible to estimate the compressibility of CNF, which is presented in Table 3. The value lies within the range of the CNF bulk modulus presented in Table 1, which shows that our estimation of droplet compressibility is correct.

If it is assumed that not all CNFs adsorb at the interface between PFP and water, ϕ can be estimated from measuring the droplet shell thickness. Figure 5 shows the transmission and scanning electron images of CNF-shelled droplets. The diameter of the droplet shown in Figure 5a was 2 µm, and by zooming in on Figure 5b showing a collapsed droplet and looking at the interface between the bright center and the darker interface, the shell thickness was estimated to be 6.5 nm. The mean diameter of several similar droplets was 2.2 µm, and the average shell thickness was 5.5 nm (Figure S1 and Table S1 in Supporting Information). It is hard to measure the exact shell thickness of droplets using TEM, rather the technique is used to estimate its value. The CNF shell is a coherent layer formed at the interface of the droplets shown in Figure 5c, where the droplet is still intact. The estimated shell thickness is approximately the size of an individual cellulose nanofiber [35], and so it is probable that the CNFs form a monolayer at the interface.

If we assume that all the other droplets are of the same size as the one measured in the TEM images (R=1.1 µm, or diameter of 2.2 µm), and that the shell thickness is constant at 5.5 nm, the calculated ϕ becomes 1.5%. This gives a more realistic higher border estimation of the volume fraction of the CNF shell, as not all CNF adsorb at the PFP–water interface. In this case, the estimated CNF bulk modulus at 240, 325, and 360 kPa was calculated to be $7.7\times 10^{10}$, $7.7\times 10^{10}$, and $7.4\times 10^{10}$ Pa, respectively. These values are higher than the ones calculated and presented in Table 3 and the ones reported in Table 1. The reason for this is that Equation (8) gives an overestimation of the true bulk modulus value [22]. If the uncertainty of shell thickness and volume fraction estimations are taken into account, the CNF bulk modulus value is similar to other values reported previously [10,36].

Three different bulk modulus values for CNF are presented in Table 1 and Table 3. One can observe that the values for CNF bulk modulus presented in Table 3 falls within the range of values presented in Table 1. On the other hand, when it is not assumed that all CNFs adsorb at the interface, the bulk modulus estimations become significantly higher. Equation (8) gives an overestimated value of bulk modulus in the direction parallel to the fiber and perpendicular to the interface surface [22], so this in combination with a probable overestimation of ϕ means that the true CNF bulk modulus for the droplet shell lies somewhere in between these extremes.

### 4.4. Acoustic Streaming

The flow profile of the acoustic streaming is presented in Figure S3 in Supporting Information. The mean value of the streaming velocity in the direction of the USW was calculated based on the flow profile (Figure S3 in Supporting Information). The absolute value of the streaming velocity of each region was multiplied by its area and then divided by the total area of the viewed channel. This velocity was then compared to the mean droplet velocity measured at the same acoustic pressure. The mean streaming velocity in the direction of the USW (y-direction) at 360 kPa was 0.5 µm/s. The mean droplet velocity at the same acoustic pressure was 2.6 µm/s. This means that the streaming velocity on average is 20% of the droplet velocity.

The y-components of the velocities in this flow (i.e., in the direction of the acoustic radiation force) varied both in magnitude and direction along the channel (x-direction). This means that although the acoustic streaming velocities constituted on average 20% of the tracked droplet velocities caused by the acoustic radiation forces, the influence of acoustic streaming on the droplet velocities was still limited. In other words, depending on the initial position of droplets in the channel, the measured droplet velocities could be both under- and overestimated. It should also be noted that the theoretical crossover particle size (diameter) between acoustic streaming and acoustic radiation force dominance is between 4 and 6 µm at 2.4 MHz and for the measured acoustic contrast factors [37]. The droplets used in our experiments had an average size similar to this theoretical crossover size, which supports our experimental observations. In future studies, the influence of acoustic streaming on acoustophoretic droplet manipulation should be analyzed in further detail. For example, by increasing the frequency to about 10 MHz, the crossover diameter would be about half the average droplet diameter, and then acoustic streaming would play a minor role.

## 5. Conclusions

In this study, the compressibility of CNF-stabilized PFP droplets was estimated using an acoustofluidic setup. The compressibility was calculated at three different acoustic pressures below the vaporization threshold. The compressibility of droplets was higher than for water but lower than for pure PFP. The decrease in compressibility was due to presence of stiff CNF at the water/PFP interface. The effects of acoustic streaming on droplet velocities were measured by particle image velocimetry using 0.5 µm and 1 µm beads. The direction of this flow varied in both direction and magnitude along the channel, which possibly resulted in an increase in uncertainty of the measured droplet compressibility. Although we could not accurately quantify the uncertainty in measured compressibility, we still demonstrated that it is possible to trap a negative-contrast-factor particle in pressure antinodes close to the bottom of a microchannel. The unique combination of high density and high compressibility makes these droplets suitable for novel applications. The findings in this study can help develop imaging sequences that enable the visualization of droplets prior to drug delivery in ultrasound-mediated therapy. Future work could focus on studying cell–droplet interaction in vitro and developing an ultrasound imaging sequence for in vivo visualization of droplet vaporization and drug release.

## Figures and Tables

**Table 1:** Material properties at 25 ${}^{\circ }$C. Tabulated values for PFP obtained from the manufacturer. The phase velocity of CNF is highly dependent on the orientation, as CNF is highly anisotropic [23].

Material	ρ [kg·m^−3^]	*c* [m·s^−1^]	κ ($1/\rho c^{2}$) [Pa^−1^]	Bulk Modulus (1/κ) [Pa]
Water	1000	1500	4.4 × 10^−10^	-
Perfluoropentane	1630	477 [24]	2.7 × 10^−9^	-
Polyamide	1030 [25]	2660 [25]	1.4 × 10^−10^	-
Cellulose nanofibers	1500 [23]	1750–3450 [23]	0.6–2.2 × 10^−10^	4.6– 17.9 × 10^9^

**Table 2:** The experimentally determined compressibilities of the Pickering-stabilized droplets at three different acoustic pressures and the theoretically estimated and experimentally determined acoustic contrast factor ϕ using Equations (2a,b) and (3).

Acoustic Pressure [kPa]	Droplet Compressibility [Pa^−1^]	Theoretical Φ	Experimental Φ
240	$6.6\times 10^{-10}\pm 0.3\times 10^{-10}$	−0.019	−0.017
325	$6.6\times 10^{-10}\pm 0.2\times 10^{-10}$	−0.019	−0.014
360	$6.8\times 10^{-10}\pm 0.7\times 10^{-10}$	−0.034	−0.029

**Table 3:** The experimentally determined compressibilities of the Pickering-stabilized droplets at three different acoustic pressures and the theoretically estimated CNF compressibility and bulk modulus based on Equation (8) and PFP compressibility. In the theoretical estimation of CNF bulk modulus, it was assumed that all CNFs were adsorbed at the interface between PFP and water and that the CNF volume fraction was 8.8%.

Acoustic Pressure [kPa]	Droplet Compressibility [Pa^−1^]	CNF Compressibility [Pa^−1^]	CNF Bulk Modulus [Pa]
240	$6.6\times 10^{-10}\pm 0.3\times 10^{-10}$	$7.5\times 10^{-11}$	$1.3\times 10^{10}$
325	$6.6\times 10^{-10}\pm 0.2\times 10^{-10}$	$7.5\times 10^{-11}$	$1.3\times 10^{10}$
360	$6.8\times 10^{-10}\pm 0.7\times 10^{-10}$	$7.8\times 10^{-11}$	$1.3\times 10^{10}$

## Supplementary Materials

The following are available online at https://www.mdpi.com/article/10.3390/mi12121465/s1; Figure S1: How shell thickness was measured in transmission electron microscope images, Table S1: Shell thickness and diameter for each droplet, Figure S2: Transmission electron microscopy image of loose cellulose nanofibers, and Figure S3: Acoustic streaming.

## Abbreviations

The following abbreviations are used in this manuscript:

CNF	Cellulose nanofiber
PFP	Perfluoropentane
USW	Ultrasonic standing wave
ARF	Acoustic radiation force
FPS	Frames per second
TEM	Transmission electron microscopy
SEM	Scanning electron microscopy
