# Supplementary material for: Measuring the Compressibility of Cellulose Nanofiber-Stabilized Microdroplets Using Acoustophoresis
Source: Micromachines (Basel). 2021 Nov 27;12(12):1465. doi: 10.3390/mi12121465 (PMC8707857; doi:10.3390/mi12121465)
Supplement: Supplementary file 1 [file micromachines-12-01465-s001.zip › micromachines-1472412-supplementary.pdf]

Supplementary

Measuring the Compressibility of Cellulose Nanofiber-Stabilized Microdroplets Using Acoustophoresis

S1 How shell thickness was measured in transmission electron microscope images

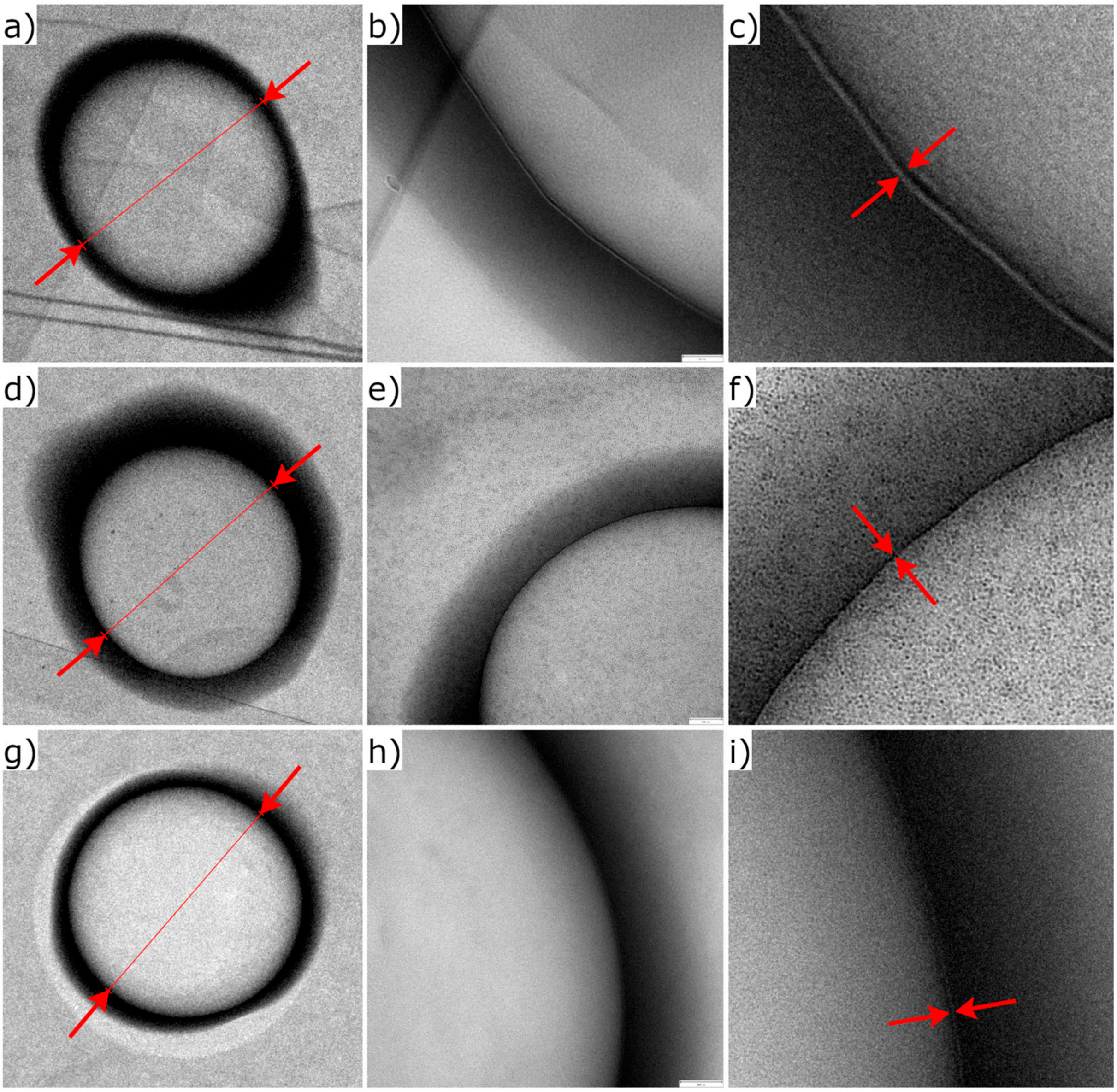

Figure S1. (**a**),(**d**) and (**g**) showed the droplets imaged with transmission electron microscopy used for shell thickness measurements. The red arrows shows between which points the droplet diameter was measured. (**b**),(**e**) and (**h**) shows the edge of the droplets imaged in (**a**),(**b**) and (**c**), respectively. The images (**c**),(**f**) and (**i**) are zoomed in regions of (**b**),(**e**) and (**h**), respectively. The red arrows in (**c**),(**f**) and (**i**) show how the shell thickness was measured.

S2 Shell thickness and diameter for each droplet

Table S1. Droplet diameter and shell thickness of droplets shown in Figure S1.

Droplet diameter [µm]	Shell thickness [nm]
2	6.5
2.3	3.8
2.3	6.2

S3 Transmission electron microscopy image of loose cellulose nanofibers

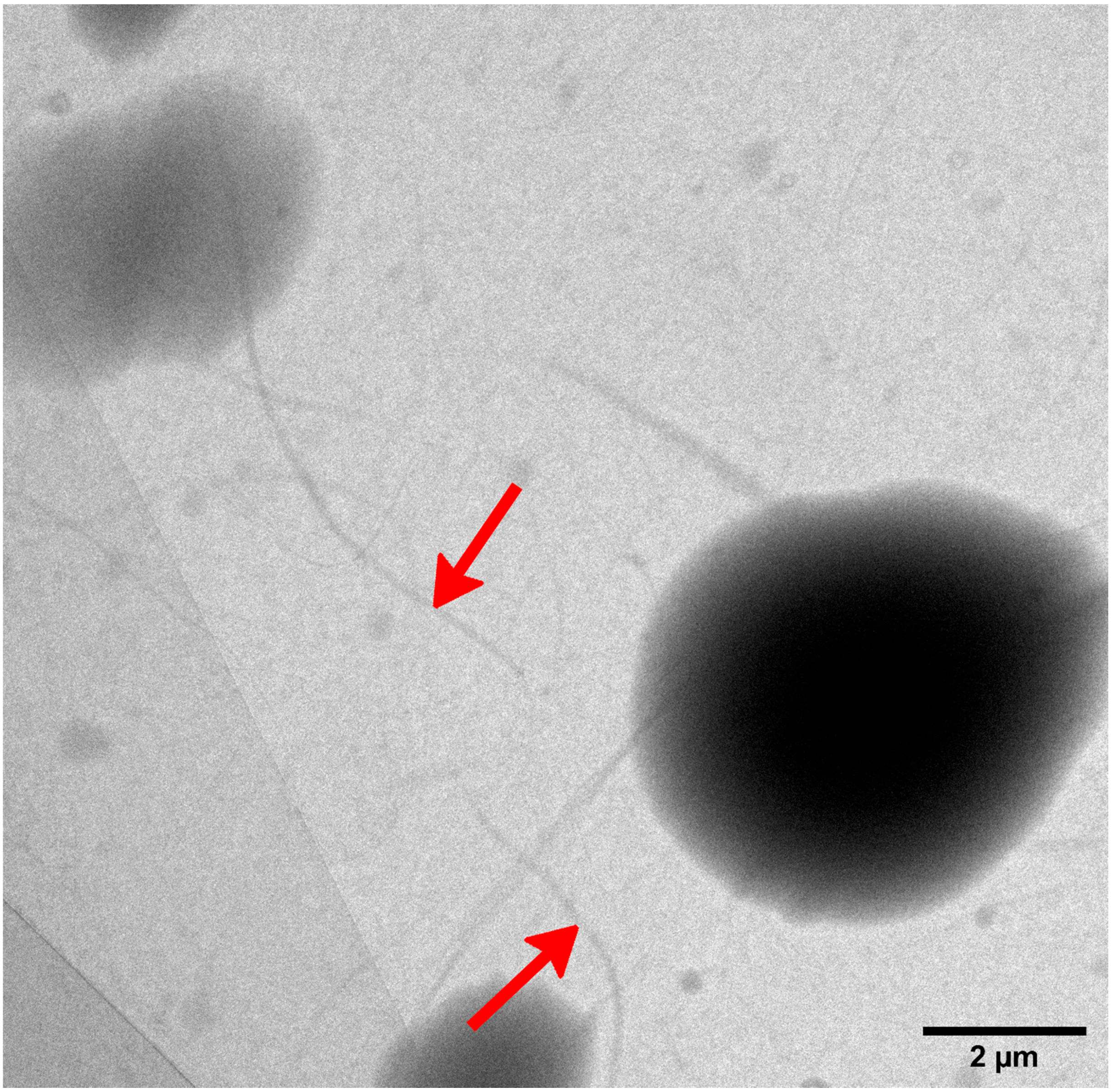

Figure S2. Transmission electron microscope image with loose cellulose nanofibers, marked with red arrows.

S4 Acoustic streaming

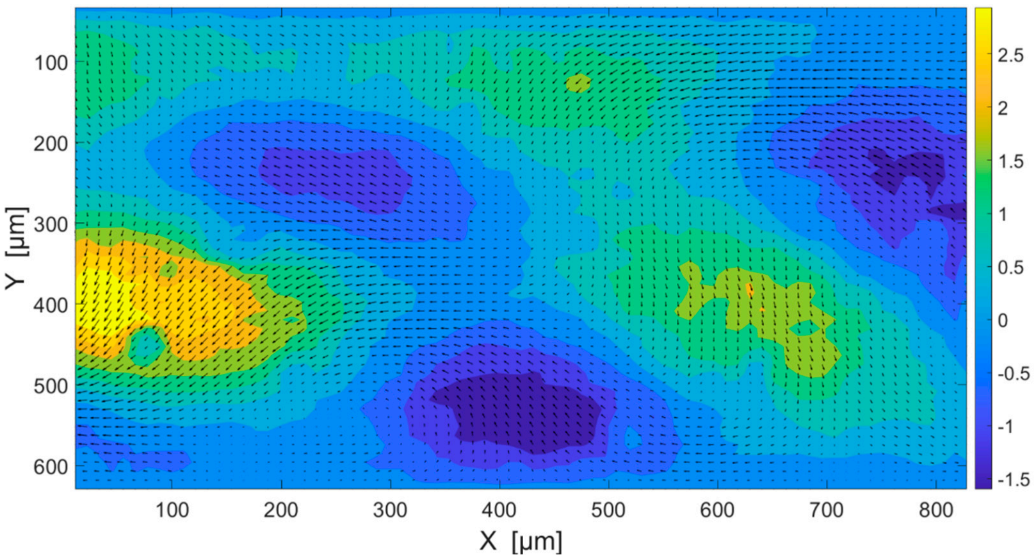

Figure S3. Ensemble correlation velocity profile of acoustic streaming measurements, performed at 360 kPa using 0.5 µm beads. The y-axis is in the direction of the microchannel width, while the x-axis is in the flow direction. The colorbar shows the velocity in the y-direction in µm/s.
